# A Case of Masturbation, with Remarks

**Published:** 1862-09

**Authors:** E. H. Neyman

**Affiliations:** Cedar Bluffs, Iowa


### CHICAGO MEDICAL EXAMINER.

N. S. DAVIS, M.D., Editor.

VOL. III. SEPTEMBER, 1862. NO. 9.

### Original Contributions.

### ARTICLE XXVI.

### A CASE OF MASTURBATION, WITH REMARKS.

By E. H. NEYMAN, M.D., Cedar Bluffs, Iowa.

I was recently called to see a patient, aged 20; found him sitting up, complaining of no other symptom than weakness, especially of the lower extremities. The closest questioning failed to elicit any other symptom. Pulse slow and languid; tongue moist and clean; respiration regular. The case puzzled me; but from a peculiar expression of his face, his inability to look me full in the face, and unsteady gait, I was lead to suspect that he was a victim of that prolific cause of evil, torture, and misery—masturbation. Victims of this habit, wishing to conceal it, answer incorrectly questions which they suppose may excite suspicion. From notes on the careful and minute examination of several cases of self-pollution, furnished me by my preceptor and *pater*, E. M. C. Neyman, I was convinced of the existance of a diagnostic sign, which from the remoteness of its situation, and the inability of the patient to discover the relation between it and his pernicious habit, would be stated without reserve or contortion. There exists, in all cases I have met, or those recorded by my father, a chronic inflammation of the testes which not only produces pain in these parts, but also a pain during violent exercise, as horseback riding, referable by the patient to the region of the external abdominal ring. I questioned my patient, indicating the region by digital pressure, ascertaining this fact, and then flatly accused him of onanism. At first he attempted to deny it, but I positively reiterated what I had said. He then confessed that he was a votary of this solitary vice; that he felt its sad influences, and at times would try to shake off the monster, but that it held him with a giant's force, his resolves at self-reform being as ropes of sand. The parents of this young man were very solicitous about his health, attributing all the difficulty to consumption. By a physical exploration of the chest, I found unmistakable evidences of phthisis pulmonalis. No hereditary predisposition could be traced. It will be remembered that BARTHEZ & RILLIET, (Diseases of Children,) place this vice in the œtiology of tuberculosis. This instance is but one of many. The vice of self-pollution is more frequent than usually imagined, numbering among its victims many of our intelligent youth. One-tenth would not be more than a fair average in the male. Of the female I am not prepared to speak positively. The effects of self-abuse are manifold and distressing, among which may be named the following: mental imbecility, nay idiocy, an awkward and irregular walk, unmanliness in the appearance, restless vision, deafness, premature gray hair, general weakness, hypochondria, impotence, decay of the spine, loss of memory, vertigo, and epilepsy. Dr. B. WOODWARD, of Galesburg, Ill., relates a case of interruption of the heart's action from this cause, (*Chicago Med. Examiner*, Vol. I., No. 3.) The pulse sank to 28, and the respiration to 5, per minute. Auscultation revealed no organic disease of the thoracic viscera. This prostrated anemic condition was sudden. As a cause of tuberculosis the authority, BARTHEZ and RILLIET have already been cited. Mr. KANE attributes some forms of night blindness to self-abuse. It is he, too, I believe, that places amaurosis as one of its sequela. MARJOLIN states, that children with a certain disease of the spinal cord, known as "Potts' disease," are usually masturbates, but whether it is a cause or an effect, he is not prepared to say. The same opinion is held by Mr. JOHNSON. If there is really any connection between the two conditions I am inclined to the conclusion, from observations on the adult, that it is but one of the many effects of onanism. The Reports of the Massachusetts Lunatic Asylum, give a percentage as high as 32 in 100, as a cause of insanity. Dr. CHIPLY, medical superintendent of the Eastern Insane Asylum, located at Lexington, Ky., in a little work, entitled "A Frightful Cause of Insanity," &c., thinks even this frightful percentage too small. GUISLAIN affirms that mental alienation, coming on without any manifest reason, has usually, in young persons, masturbation for a cause. Dr. CHIPLY enumerates dyspepsia and drunkenness among the effects. I remember of seeing, while a student of medicine, a patient who complained of continuous and distressing tinnitus aurium and phantasmascopia; stysis was impossible, the nearest approach to plenary styrna, being only semi-erection. In this disease, while the mind is impaired, the imagination is unbridled, which pencils pictures, gross, lascivious, and beastly. Nature becomes demoralized, and the victim left incapable of one pure sentiment.

Another curious, interesting, and important fact, is the early age at which this disease may be contracted. We have good authority (MARJOLIN), that even infants at the breast, fall into the habit. Dr. VAN BAMBEKE relates, in the "*Union Medicale,*" three cases in children between the ages of ten and twenty months. A. A. W. JOHNSON, F.R.S., to whom I am indebted for much valuable matter in this paper, relates an instructive case, which came under his own observation. The patient, aged about six, commenced ailing when about two years and ten months old. He became weak and ailing, without any definite malady. Slight improvement, with frequent relapses were experienced upon the administration of tonics. Finally, deafness complicated the other symptoms; the child, instead of presenting the appearance of a healthy child, became to all appearance a little dried up old man, (*vide London Lancet.*) This was first contracted by sleeping with a young girl, 14 or 15 years of age. BARTHEZ and RILLIET, in their admirable work before referred to, say, "It is often very young children that abandon themselves to it with a fury." M. MARJOLIN observed the same thing in the Hospital des Enfans Malades, (*vide Report in the Gazette*

*des Hospitaux.*) FOURNIER and BEGIN relate the case of a little girl, who died in a state of woful marasmus. ZIMMERMAN also refers to the frequency of the disease.

An author, writing on this subject, says that during the paroxysm, the face becomes flushed and moist, the child oblivious, its position reclining, and eyes unusually brilliant. After the act, there follows exhaustion, non-inclination to play, pale livid face, and a dull, expressionless eye. These remarks particularize infants. I was told by a victim of self-pollution, that during one winter, she had a sparce allowance of bed clothing, and would often wake up benumbed with cold, on such occasions, she would often send the warm blood gushing through its vessels, and warming her benumbed extremities by performing the act. She would then sink to sleep. The exercise of the generative organs at a very early period, will hasten their development. Thus in Mr. JOHNSON'S patient, there were emissions; and in one of Dr. VAN BAMBEKE'S cases, a little girl, in which the periods of excitement were very frequent, a considerable development of the erectile organs existed.

Mr. JOHNSON states that it is not absolutely necessary that the patient use his hand, but that he may effect his purpose by a "peculiar convulsive or instinctive movement of the thighs." The same author concludes that the evil consequences of infancy are greater than to adolescence or maturity. This stands to reason; the nervous system being highly excitable in infancy, will take recognizance of impression sooner than the less excitable adult. While in adults the organs and faculties being forward, it can only impair; in the child, these being only forming, it both impairs and stagnates. In proof of the greater susceptibility of children, might be adduced the fact that even adolescence falls a victim to over-legitimate sexual indulgence much sooner than maturity.

Our next inquiry would be into the causes of the habit. LALLEMAND, in his elaborate work, has a long category of causes. Dr. CHIPLY'S chapter of causes embody the same. None are more prominent than the vicious and imprudent acts of attendants and associates. Especially should care be used in placing young male infants to sleep with young girls. It will be remembered that Mr. JOHNSON'S patient contracted the disease by sleeping with a female attendant. In the same paper, he gives an illustration, the instance of a boy aged seven, who was treated by him, in the Children's Hospital, for gonorrhœa and buboes, contracted apparently while sleeping with a servant girl. The predisposing cause, Mr. JOHNSON seems to think, is the excitability of the nervous system during early life. Potts' disease has already been alluded to. Dr. VAN BAMBEKE seems to think that during dentition, where there exists an increased nervous excitability, it is frequently a cause. In males, the secretion of the *glandul odoriferi Tysoni* becomes lodged in the *fossa glandis*, behind the *corona*, producing *pruritis*, leading to onanism. Dr. CHURCHILL, in his work on Diseases of Women, gives pruritis of vulva as a cause of self-abuse, and consequently, indirectly, anything that will produce this, whether the aphthous inflammation of DEWEES' (Diseases of Fem., p. 47,) disease of the membraneous lining of the womb, of BLUNDELL, (Diseases of Woman,) superabundance of hair on external genitals, of DAVIS' (*vide Obst. Med.* Vol. I.) diabetes, of WEST, (Sec. Dis. of Fem.) lichen, prurigo and eczema, of FOURNIER, (*L'Union Medicale*, 1851,) *ascaris virmicularis in rectum*, circumscribed inflammation of vulva, or the accumulated acrid secretion of the sebaceous glands. Dr. DAVIS, in his work on Obstetric Medicine, gives enlarged clitoris as a cause. LALLEMAND, among other things, enumerates late rising, feather beds, diseases of bladder, and elongated prepuce. In reference to the latter, Dr. COPLAND says: "The neglect of circumcision, in Christian countries, is certainly no mean physical cause of the prevalence of this vice, and of the many consequences which follow." I think I may venture on the assertion, that in adult females, inflammation and ulceration of the mouth of the uterus, is sometimes a cause. Sometimes the means we employ, expecting to do the greatest good, are instruments of death; as, for instance, one patient told me that he first contracted the vice by reading a book, cautioning him against it. He said the author portrayed, in such emphatic terms, the impossibility of resisting the desire, that he naturally concluded that the pleasure must be very great. He thought he would try it once, "to see how it would feel." The result was, he became a confirmed masturbator. These remarks apply equally well to opium eating; I know one physician, an opium eater, who contracted the habit by the emulative desire of witnessing the effect of medicines upon his own person. Popular medical education is a humbug. This is the only objection I have to Dr. CHIPLY'S otherwise admirable little volume.

It is often important that we should be able to diagnose these cases beyond cavil. In youth and adults too much importance cannot be attached to the pain referred by the patient to the external abdominal ring. In connection with the blushing, unmanly awkwardness, and unsteady gait and vision, it is almost certain. Where melancholy alternates with sudden and unexplained exuberance of mirthfulness; or imbecility with good cranial conformation, suspicion should be aroused. I have found, or imagined I have found, an unusual prominence of the os pubes, from a superabundance of adipose tissue. This, together with frequent erection, causes an unseemly prominence of the parts in the region of the genitals. This may form a valuable co-index in connection with other indices. There is also, usually, a pimple on the face, the gutta rosea, very characteristic. I never see continual successive crops of these, especially in the male, but what my suspicions are aroused. In young infants, there is usually no tendency to hide it, but it is unblushingly performed. In older children, Dr. DONNE'S test may be used. This gives chrystals of the oxalate of lime, upon an analysis of the urine. Especial care should be observed, in all our examinations, that we may not learn the object of our scrutiny the vice, if he has not already contracted it. In any thing like young children, "moral suasion" will prove futile, for no child, in consideration of all the suffering, in the future, you may be able to picture to the youthful mind, will surrender a present pleasure.

The causes, symptoms, and diagnosis having been given in detail, the most vague and unsubstantial part yet remains to be spoken of; namely, the treatment. It is here that the physician, in disease, has too often to stop in his investigation, unable to go farther, to accomplish which, all the rest were but subsidiary. The top round in the ladder is missing, all the previous ascent is useless, for the grand object—a cure—has not been effected. Thus the pathologist may successively trace the symptoms, causes, diagnosis, and pathological anatomy of chronic hydrocephalous, epilepsy, or Bright's disease of the kidney, but if he fails in arriving at some mode of treatment, his labors have been comparatively useless. We must confess, that without the patient's aid such is nearly the position of an investigator of onanism.

As a general indication of treatment, I would say, remove the cause, as the first step; viz.: if elongated prepuce, circumcise; if hyperthrophied clitoris, amputate; if sebaceous secretion within prepuce, remove it; if pruritis, cure it; if from dentition, hasten it as much as possible; if diabetes, treat that, etc. The simple removal of the cause will be found inadequate to a cure, after the pernicious habit has once got a fair grip of the patient, who is either too young to understand how imminent his danger is, or has too little self-control to discontinue a habit. The one case will have to be restrained, in the other, the patient must be assisted to conquer that which he is insufficient alone to do. If we possessed medicines powerfully antiphroditic, either would be easy of accomplishment. But none such are embraced in our known therapeutical agents. The next best mode of cure is the establishing of an irritation, which will prevent, from pain, the use of friction. It is said that, in young children, the sudden dashing of water on the parts, while in the height of excitement, will sometimes, from the shock produced, effect a cure. It will be found most convenient in early age to pare the prepuce, place the child on a hard bed, learn it to sleep with its hands without the cover, and to enjoin a close surveillance, both night and day. This will usually be found sufficient; if not, irritation may be kept up by means shortly to be described. In grown males, if they will submit, the prepuse may be excised, if not, epispastics should be employed. The best vesicant I have found is croton oil, which does not possess the disadvantage of cantharides, which are liable to produce priapisms and strangury; or of sinapisms, which produce a blister, instead of distinct and interrupted vesicles or pustules. These may be kept open by either unguentum cantharidis, or unguentum antimonii. I have frequently thought that if an impervious vagina, incapable of transmitting impressions, could be so constructed as not to incapacitate micturition, that it would afford material aid in assisting the patient in breaking loose from this vice. My idea is vague and flippant. I would have a belt pass around the body and fasten by lock and key, as in anchylosis apparatus. In grown females, the general indication to remove the cause should be followed, and then if an educated female conscience, and an innate female modesty and purity does not restrain them, they *are not worthy of treatment.*

And now I come to speak of *dernier resort.* It is well known that castration has been frequently resorted to, in the cure of epilepsy, having masturbation as a cause. When all other means fail, and epilepsy, idiocy, or insanity are imminent, I would unhesitatingly advise castration.

Before we close, there is one interesting point in the discussion of onanism, which is well worth a moment's consideration. The victim of self-abuse often quiets his conscience by asking why moderate onanism should be productive of more mischief than an equally moderate amount of legitimate indulgence? Is it because of the morbid excitement of the mind, the humiliation of an educated and intelligent conscience, or as some fanciful empiric suggests, may not there exist two kinds of electricities, which at the acme of excitement mutually replace each other, establishing an equilibrium, during normal sexual intercourse, but that during masturbation but one kind of electricity alone is expended? Dr. W. Goodell, member of the Imperial Society of Constantinople, in an article entitled "Turkey from a Medical Point of View," says:—"Neither onanism nor pedarastry, both of which prevail to a frightful extent in the East, emasculate the *morale* of the Oriental as much as they do the European." The restlessness, unmanliness, and unsteady vision are not seen. In explanation of this anomaly, he thinks we may draw one or two inferences; namely, that it should be attributed more to the "humiliation of an educated conscience than any physical drain from the system; or where society openly upholds a vice it is not so apt to be intemperately abused, as when local rules of propriety, and militant precepts of religion compels the votary to invest the indulgence in strict privacy," (*vide N. A. Med. J.*, Vol. V., No. 3.)

